# Mapping Temporally Ordered Inputs to Binary Message Outputs with a DNA Temporal Logic Circuit

**DOI:** 10.3390/nano13050903

**Published:** 2023-02-27

**Authors:** Shuai Zhao, Yuan Liu, Xiaokang Zhang, Rui Qin, Bin Wang, Qiang Zhang

**Affiliations:** 1 Key Laboratory of Advanced Design and Intelligent Computing, Ministry of Education, School of Software Engineering, Dalian University, Dalian 116622, China; 2 School of Computer Science and Technology, Dalian University of Technology, Dalian 116024, China

**Keywords:** DNA nanotechnology, DNA strand displacement, DNA temporal logic circuits, chemical reaction networks, molecular encryption

## Abstract

Molecular circuits and devices with temporal signal processing capability are of great significance for the analysis of complex biological processes. Mapping temporal inputs to binary messages is a process of history-dependent signal responses, which can help understand the signal-processing behavior of organisms. Here, we propose a DNA temporal logic circuit based on DNA strand displacement reactions, which can map temporally ordered inputs to corresponding binary message outputs. The presence or absence of the output signal is determined by the type of substrate reaction with the input so that different orders of inputs correspond to different binary outputs. We demonstrate that a circuit can be generalized to more complex temporal logic circuits by increasing or decreasing the number of substrates or inputs. We also show that our circuit had excellent responsiveness to temporally ordered inputs, flexibility, and expansibility in the case of symmetrically encrypted communications. We envision that our scheme can provide some new ideas for future molecular encryption, information processing, and neural networks.

## 1. Introduction

In nature, how living systems process internal and external signals is complicated and diverse and depends on the complexity, type, and temporal order of the signals. Living organisms respond to variable identities and orders of signals with their internal temporal regulatory networks and thus exhibit diverse behaviors and phenotypes [1]. For example, transcription factors trigger different gene expressions under different temporal schedules in response to stress [2]; mutations’ order affects the formation and evolution of myeloproliferative neoplasms [3]; and regulation of gene networks in temporal patterns can expand neuronal diversity [4]. It can be seen that responsiveness to temporal signals is critical for organisms. Similarly, designing synthetic molecular devices and molecular circuits that can unveil or process underlying temporal information could have a wide range of practical applications in the biological field [5].

In prior studies, several schemes have been proposed to respond to temporally ordered inputs [6,7] and signals [8,9]. Many measures of implementation have been designed for different purposes. For example, two-ring DNA [6] can be used for sensing by recognizing the order of input; the DNA strand displacement reaction [7] determines the winner of tic-tac-toe by taking different events with different input order. Notably, DNA strand displacement technology [10,11,12] is considered an excellent reaction interface in chemistry [13,14], materials [15,16], and medicine [17,18] due to its universal chemical kinetic system [19,20,21] and ability to perform complex calculations [22,23]. Specifically, DNA strand displacement mechanisms have been widely used to build various synthetic molecular systems, such as logic circuits [24,25,26], nanorobots [27,28,29], and neural networks [30,31,32]. In addition, the DNA strand displacement reaction does not require the assistance of enzymes, which can help avoid some practical problems. For example, some enzymes are susceptible to the reaction environment or conditions, which may increase the complexity and cost of the experiment [33]. Importantly, the DNA strand displacement technique alone also responds well to temporally ordered inputs. For example, an addressable activated cascade DNA sequential logic circuit [34] can respond to the order of three inputs, and a DNA strand displacement temporal logic circuit [35] can recognize the different order of two inputs. Moreover, the latter circuit has been proven to be generalizable to more complex temporal logic circuits to respond to more inputs. Such temporal logic circuits with expansibility enable time-dependent learning rules, which may guide the future intelligence of artificial molecular machines [35].

Inspired by this idea, we propose a scheme for DNA temporal logic circuits based on DNA strand displacement reactions. According to our design rules, our circuits can be generalized to larger-scale temporal logic circuits by varying the number of substrates or inputs. Specifically, we studied the mapping relationship between the temporally ordered inputs and the binary message when the number of inputs and substrates is different. Here, we present the corresponding simulation model. The circuit was applied to the symmetric encryption of binary messages to demonstrate the effectiveness of mapping temporally ordered inputs into binary messages. We constructed a five-input, five-substrate DNA temporal logic circuit using a simulation model and used the circuit as the key for encryption and decryption. Finally, we used this circuit to demonstrate a case of symmetrically encrypted communication of binary messages to show its responsiveness, flexibility, and expansibility.

## 2. Materials and Methods

### 2.1. Materials

All DNA samples used in this paper were purchased from Sangon Biotech. Co., Ltd. (Shanghai, China). The unmodified DNA strands were purified by polyacrylamide gel electrophoresis. DNA strands modified with a fluorophore or quencher were purified by high-performance liquid chromatography. The fluorophores of the three modifications were FAM, ROX, and CY5.5, respectively, and the corresponding quenching agents of the three modifications were BHQ1, BHQ2, and BHQ3, respectively. The concentration of DNA molecules was measured using the NanoDrop 2000 spectrophotometer (Thermo Fisher Scientific Inc., Waltham, MA, USA) at an absorbance of λ = 260 nm.

### 2.2. Annealing of Substrates

All substrates were dissolved as single strands of DNA at the same concentration (3 µM) in 50 µL of 1 × TAE/Mg^2+^ buffer (40 mM Tris, 20 mM acetic acid, 1 mM EDTA 2Na and pH balanced to 8.0) and placed inside a polymerase chain reaction (PCR) thermocycler to anneal. The temperature was set to be heated at 95 °C for 10 min, cooled to 25 °C at the rate of 1 °C/min, and maintained at 25 °C for 10 min.

### 2.3. Native PAGE Characterization

All samples were dissolved in 30 µL of 1 × TAE/Mg^2+^ buffer and reacted for 2 h at 37 °C. The samples to be tested were mixed with 3 µL of 50% glycerol solution and analyzed in a 12% native polyacrylamide gel after running at a constant pressure of 85 V for 120 min.

### 2.4. Fluorescence Kinetics

All fluorescences were measured using a real-time PCR system (Bio-Rad, CFX96, Bio-Rad Laboratories, Inc., Hercules, CA, USA). All samples were added to 50 µL of 1 × TAE/Mg^2+^ buffer at 37 °C for fluorescence analysis. All fluorescence experiments were repeated more than three times to ensure reproducibility.

## 3. Results

### 3.1. The Principle of Our DNA Temporal Logic Circuit

**Figure 1:**
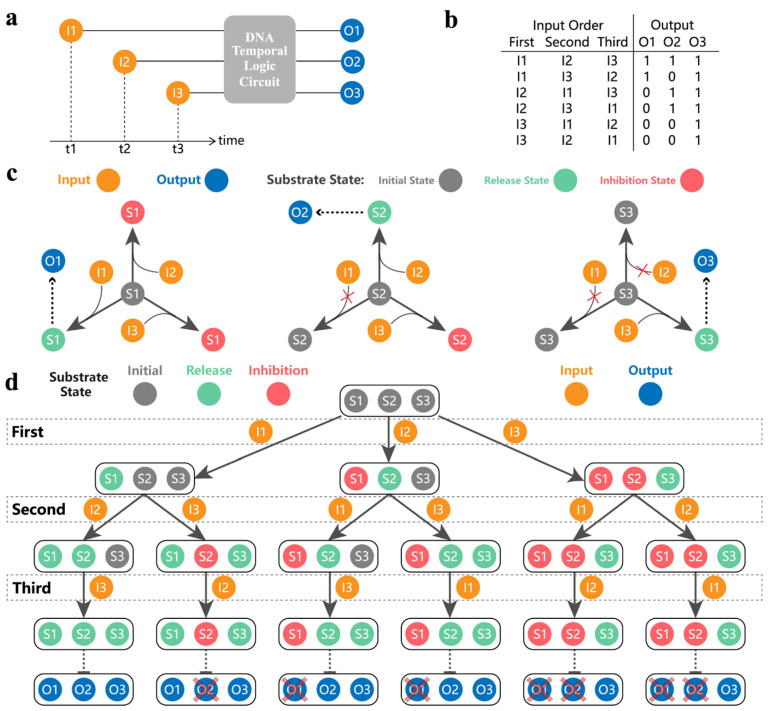
The principle of our DNA temporal logic circuit. (**a**) Abstract circuit diagram. I1, I2, and I3 represent input1, input2, and input3, respectively. O1, O2, and O3 represent output1, output2, and output3, respectively. (**b**) The mapping relationship between temporally ordered inputs and binary message outputs. (**c**) The basic principle of the DNA temporal logic circuit. Orange represents the inputs. Blue represents the outputs. S1, S2, and S3 represent Substrate1, Substrate2, and Substrate3, respectively. The substrate state is gray (initial state), green (release state), and red (inhibition state). How the three substrates respond to each input is sequentially arranged from left to right. (**d**) The response process of the DNA temporal logic circuit under different orders of inputs.

A DNA temporal logic circuit is defined in Figure 1a. For ease of presentation, input1 is abbreviated as I1, output1 is abbreviated as O1, and so on. The circuit can produce different outputs when the combinations of inputs are the same but in different temporal orders. The mapping relationship between the temporally ordered inputs and the binary message outputs is shown in Figure 1b. Notably, in our circuit, the same input order has the same reaction path. For example, for a reaction system with only three inputs, when the input order is I1, I3, and I2, the reaction path is that the substrates react first with I1, then with I3, and finally with I2. However, with I1 and I3 as the input order, the reaction path is that the substrates react with I1 first and then with I3. It can be found that when the input order is I1, I3, and I2, the reaction path contains the reaction path that occurs when the input order is I1 and I3. In other words, the reaction path when all three inputs are present contains the reaction path which occurs when some input is absent. Therefore, we focused on the order of the inputs when all three inputs are present and did not study the order of the inputs when one or more inputs are absent to prevent repeated validation.

Figure 1c shows the basic principle of implementing a DNA temporal logic circuit. Different inputs and outputs are distinguished according to different letters and subscripts. Here, we define three states of the substrates, namely, the initial state, the release state, and the inhibition state. In the beginning, all substrates are in the initial state. When the subscript of the input is equal to the subscript of the substrate (such as I1 and S1, I2 and S2, and I3 and S3), the substrate can undergo a release reaction and change from the initial state to the release state. This process produces output, and the substrate of the release state does not respond to any subsequent inputs. When the subscript of the input is greater than the subscript of the substrate (such as I2 and S1, I3 and S1, and I3 and S2), the substrate can undergo an inhibition reaction and change from the initial state to the inhibitory state. This process produces no output, and the substrate of the inhibition state does not respond to any subsequent inputs. When the subscript of the input is less than the subscript of the substrate (such as I1 and S2, I1 and S3, and I2 and S3), the input does not react with the substrate. The state of the substrate remains unchanged and is converted to the release or inhibition state depending on the subsequent trigger. According to this design principle, when the three substrates are mixed together, different temporally ordered inputs can be mapped to different output results. The specific process is shown in Figure 1d. We assume that all inputs are in excess. Take the order of inputs I2, I1, and I3 as an example. When I2 is input for the first time, I2 can change S2 of the initial state to the release state and can change S1 of the initial state to the inhibition state. The two reactions occur at the same time; so, S1 turns into a red inhibition state and S2 turns into a green release state. When I1 is input for the second time, S1 is inhibited and does not react with I1 so the state of all substrates does not change. When I3 is input for the third time, I3 has a release reaction with S3, which turns S3 into the green release state. Finally, the substrates in the released state are S2 and S3. The release state means that the substrate has undergone a release reaction to produce the output; so, the output produced by the whole process is O2 and O3. The binary message is (0,1,1), which corresponds to the mapping relationship of Figure 1b. It can be seen that the principle can map temporally ordered inputs into binary messages.

**Figure 2:**
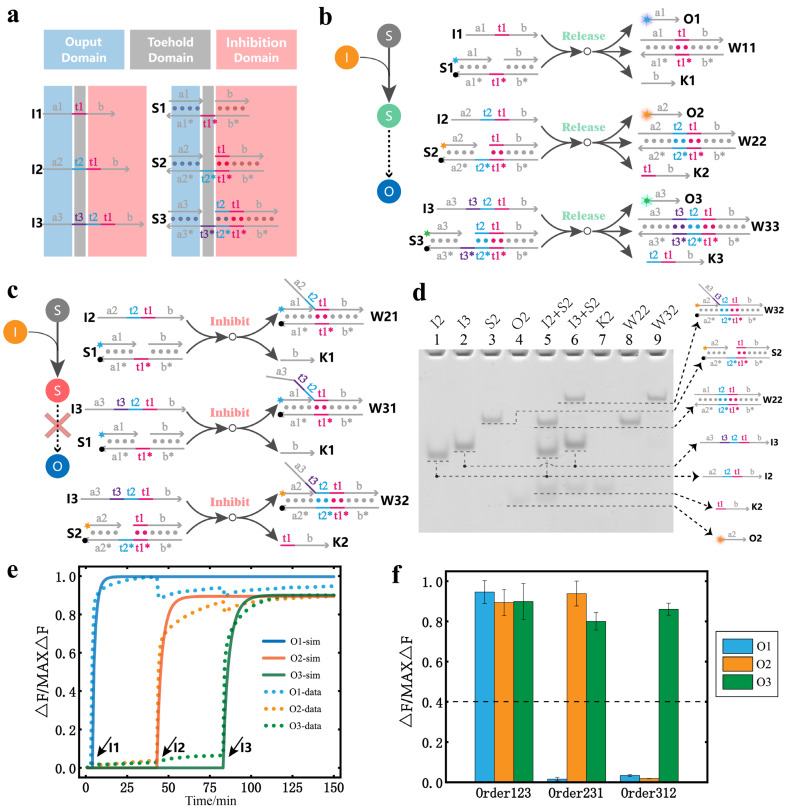
Design and implementation of the DNA temporal logic circuit. (**a**) The design of the DNA temporal logic circuit. Blue represents the output domain; gray represents the toehold domain; red represents the inhibition domain. (**b**) The strand displacement reaction process for all release reactions. (**c**) The strand displacement reaction process for all inhibition reactions. (**d**) Polyacrylamide gel electrophoresis was used to analyze the release reaction and inhibition reaction of S2. The top of the gel was marked with the reference of the reactant, the reference of the product, the results of the release reaction, and the results of the inhibition reaction of the substrate S2 ([I1] = [I2] = [I3] = 2400 nM, [S2] = 800 nM). (**e**) Fluorescence kinetics. S1, S2, and S3 were added to the solution before input. The reaction temperature was 37 °C, and the frequency was 1 data point per minute. The order of the input was I1, I2, and I3, and the time interval of the input was 40 min ([S1] = [S2] = [S3] = 120 nM, [I1] = [I2] = [I3] = 400 nM). (**f**) Bar graph of the output fluorescence signal with different orders of inputs. The dashed line marks the separation between the output results 0 and 1. All the asterisks in the figure indicates the complementary strand of the corresponding strand.

To implement the above principle of mapping temporally ordered inputs to binary messages, we designed the circuit shown in Figure 2a using the DNA strand displacement reaction. All inputs and substrates were composed of three kinds of domains, namely, the output domain, toehold domain, and inhibition domain. All inputs were designed as single strand DNA. All substrates could undergo DNA strand displacement through their own unique toehold domain. Moreover, the inputs and substrates with the same subscript have the same output domain, toehold domain, and inhibition domain. The toehold and inhibition domains of the existing input and substrate are combined to form the inhibition domain of the next subscript input and substrate. According to these design rules, when the subscript of the input is equal to that of the substrate, the corresponding input and the substrate can undergo a DNA strand displacement reaction. At the time of producing the output, a stable double strand structure can be generated so that it no longer responds to other subsequent inputs and becomes a release state. The specific process of the release reactions is shown in Figure 2b. When the subscript of the input is greater than the subscript of the substrate, the input can also undergo a strand displacement reaction with the substrate. However, there was no output after the reaction due to different output domains. The generated double strand structure is relatively stable; so, it does not respond to other subsequent inputs and becomes an inhibition state. The specific process of the inhibition reaction is shown in Figure 2c.

All the release and inhibition reactions are similar DNA strand displacement reactions; so, we illustrate only the release and inhibition reactions of S2 in Figure 2d. As shown in the experimental results of the polyacrylamide gel electrophoresis, lanes 1, 2, and 3 are the reference for the reactants. Lanes 4, 7, 8, and 9 are the reference for the products. Lane 5 represents the results after the release reaction between I2 and S2. Lane 6 represents the results after the inhibition reaction between I3 and S2. Because the inputs are excessive, there is residual I2 and I3 in lanes 5 and 6, respectively. In addition, there is the presence of O2 and K2 and the formation of the stable duplex structure W22 in lane 5, which indicate that a release reaction has occurred. In lane 6, the absence of O2, the presence of K2, and the formation of the stable duplex structure W32 indicate that an inhibition reaction has occurred.

The results of circuit mapping the temporally ordered inputs to binary messages are indicated by the fluorescence experiments shown in Figure 2e, which shows the real-time fluorescence curve and simulation curve when the order of inputs is I1, I2, and I3. The input time interval is 40 min. It can be seen that with the addition of I1, I2, and I3, the fluorescence intensity of O1, O2, and O3 increased accordingly. All simulation models and reaction rate constants are given in the Supplementary Material (Supplementary Section S3). To express the output results more clearly, we present the fluorescence results in the bar graph shown in Figure 2f. The inputs in the order I1, I2, and I3 are abbreviated as, for example, Order123. It can be seen that when the input is in Order123, no signal is inhibited, and the binary message result of the mapping is (1,1,1). When the input is in Order231, the signal of O1 is mostly inhibited and the binary message result of the mapping is (0,1,1). When the input is in Order312, the signals of O1 and O2 are mostly inhibited and the binary message result of the mapping is (0,0,1). The results show that our design enables DNA circuits to respond to different temporally ordered inputs. The fluorescence results for the other input orders are provided in the Supplementary Material (Supplementary Figures S3 and S4).

### 3.2. The Expansibility of Our DNA Temporal Logic Circuit

The DNA temporal logic circuit constructed above is a reaction system with three inputs and three substrates. The design rules and responsiveness to mapping temporally ordered inputs to binary messages have been described. Notably, a larger-scale temporal circuit with more inputs and substrates can be easily extended according to our design rules. Theoretically, a DNA circuit with any arbitrary number of *n* inputs and *n* substrates can be designed by increasing or decreasing the number of inputs and substrates. Therefore, we studied the circuit when the number of inputs and the number of substrates changed.

#### 3.2.1. Changes in the Number of Substrates

**Figure 3:**
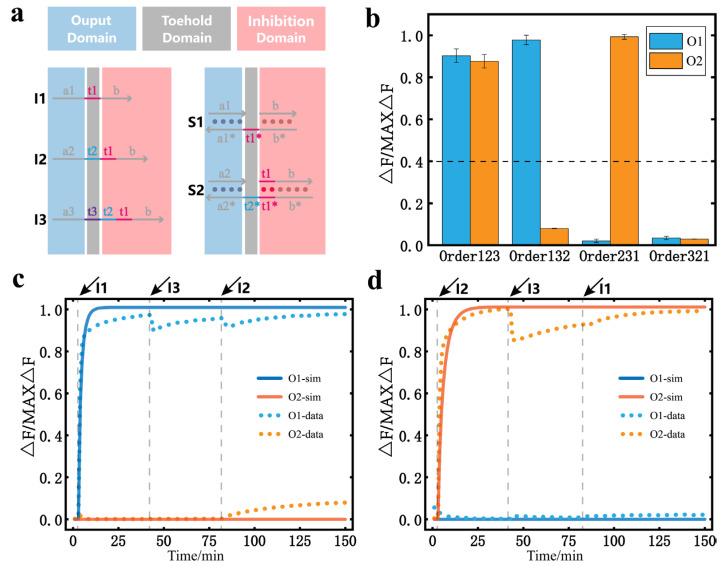
DNA temporal logic circuit with three inputs and two substrates. (**a**) Design of the temporal logic circuit with three inputs and two substrates. (**b**) Bar graph of the output fluorescence signal with different orders of inputs. The dashed line marks the separation between the output results 0 and 1. (**c**) The curve of the fluorescence data and simulation data when the order of inputs is I1, I3, and I2, and the interval of input addition is 40 min ([S1] = [S2] = [S3] = 120 nM, [I1] = [I2] = [I3] = 400nM). (**d**) The curve of the fluorescence data and simulation data when the order of inputs is I2, I3, and I1, and the interval of input addition is 40 min ([S1] = [S2] = [S3] = 120nM, [I1] = [I2] = [I3] = 400nM). All the asterisks in the figure indicates the complementary strand of the corresponding strand.

When the number of substrates was changed, we first explored the temporal logic circuit of three inputs and two substrates. The design of the specific circuit is shown in Figure 3a. Because there are only two substrates, the corresponding output signals are O1 and O2. The binary message mapped by the temporally ordered inputs is different from the previous circuit. Notably, there are four combinations of two bits of binary numbers, which are (0,0), (0,1), (1,0), and (1,1). The results in Figure 3b correspond to all four of these binary outputs. It can be found that in the temporal logic circuit of three inputs and two substrates, the result of O3 in any result is always 1. Because there is no input that can inhibit S3, I3 eventually undergoes a release reaction with S3. However, for the circuit with three inputs and two substrates, each substrate can be released or inhibited. The corresponding output results can be 0 or 1; so, the circuit can contain all combinations of two bits of binary messages. In fact, for a circuit with n + 1 inputs and n substrates, it is also guaranteed that each substrate is eventually in the release or inhibition state. In other words, the output of such a circuit has a total of 2n cases, including all n-bit binary number combinations, which means that it can ensure the integrity of n bits of binary messages when mapping. The real-time fluorescence curves of the reaction process with Order132 and Order231 are given in Figure 3c,d. The fluorescence results for the other input orders are provided in the Supplementary Material (Supplementary Figures S1 and S2).

#### 3.2.2. Changes in the Number of Inputs

**Figure 4:**
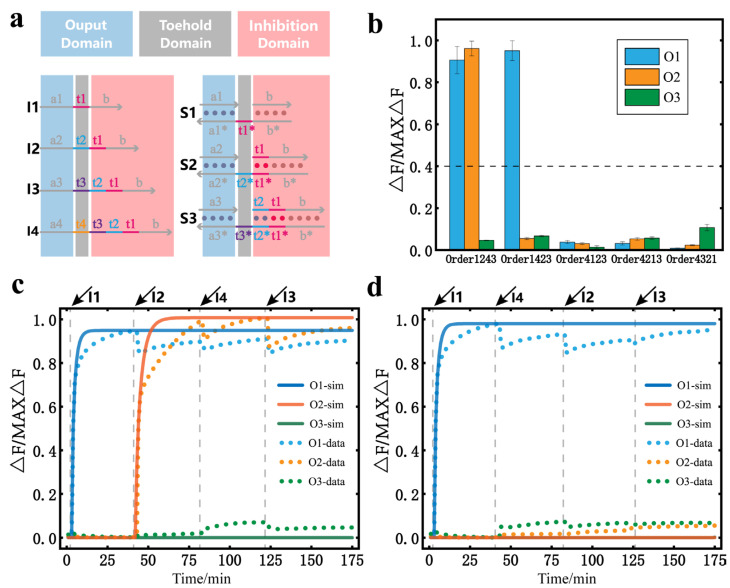
DNA temporal logic circuit with four inputs and three substrates. (**a**) Design of the temporal logic circuit with four inputs and three substrates. (**b**) Bar graph of the output fluorescence signal with different orders of inputs. The dashed line marks the separation between the output results 0 and 1. (**c**) The curve of the fluorescence data and simulation data when the order of inputs is I1, I2, I4, and I3 and the interval of input addition is 40 min ([S1] = [S2] = [S3] = 120 nM, [I1] = [I2] = [I3] = 400 nM). (**d**) The curve of the fluorescence data and simulation data when the order of inputs is I1, I4, I2, and I3 and the interval of input addition is 40 min ([S1] = [S2] = [S3] = 120 nM, [I1] = [I2] = [I3] = 400 nM). All the asterisks in the figure indicates the complementary strand of the corresponding strand.

We explored the temporal logic circuit with four inputs and three substrates. The binary messages of the temporally ordered inputs map are also different from all previous circuits. The design of the specific circuit is shown in Figure 4a. Because S3 is the substrate with the largest subscript, I4 can only inhibit other substrates. Here, we only studied the inhibitory effect of I4. The corresponding output signals for different orders of inputs are shown in Figure 4b. It can be seen that after the addition of I4, the substrates in the initial state all change to the inhibition state, and no more output is produced. In particular, when I4 was used as the first input, the remaining three inputs failed to release the corresponding output signal regardless of the order in which they were added, proving that I4 had a significant inhibitory effect. Based on the inhibitory effect of I4 and the mapping relationship of the previous three inputs and three substrates circuit, we can assume that all input orders are capable of mapping 23 different binary outputs in this circuit. The real-time fluorescence curves of the reaction process with Order1243 and Order1423 are given in Figure 4c,d. The fluorescence results for the other input orders are provided in the Supplementary Material (Supplementary Figures S5 and S6).

### 3.3. Application to the Symmetrically Encrypted Communication of Binary Messages

DNA temporal logic circuits are essentially the mapping of different orders of inputs to specific binary messages. According to our design rules, more inputs can be mapped by varying the number of inputs and substrates. Theoretically, the expansibility allows the number of binary bits output to increase arbitrarily. The n + 1 inputs n substrates circuit can ensure the integrity of n bits of binary messages when mapping. This means that any binary message we want to express can be included. Therefore, we propose to encrypt and decrypt binary messages through our scheme. According to the explicit mapping relationship between the input order and the output, the timing input is equivalent to a binary message when transmitted. Specifically, we created a five-input, five-substrate temporal logic circuit using a simulation model and used the model to demonstrate the case of encrypted communication with binary messages.

**Figure 5:**
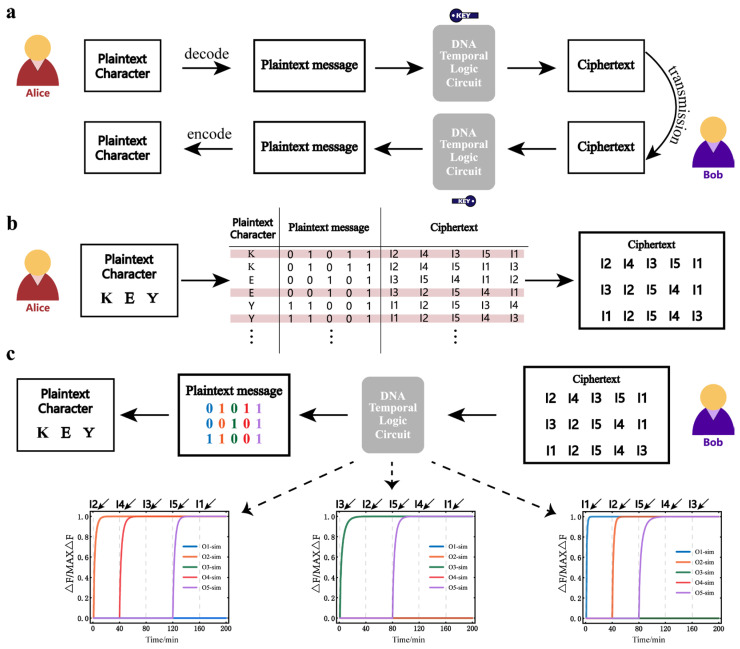
Application to the symmetrically encrypted communication of binary messages. (**a**) The information communication process between Alice and Bob. DNA temporal logic circuits are used as the key for symmetric encryption. (**b**) Alice encrypts the plain text into the cipher text according to the cipher book (the mapping table of the DNA temporal logic circuit). Alice has a variety of encryption options. She chose only the three cases in the red line. (**c**) The process of Bob’s decryption.

The information communication process of the sender, Alice, and the receiver, Bob, is shown in Figure 5a. Alice first encodes plain text characters into a binary message (plain text message). The specific encoding and decoding process is given in Supplementary Materials Table S3. The plain text message is then encrypted into the cipher text according to the mapping relationship of the temporal logic circuit of the five-input, five-substrate circuit. The specific operation of Alice is shown in Figure 5b. It can be seen that Alice encrypts the plain text characters into the cipher text according to the cipher book. Here, Alice has a variety of encryption options. She chose only the three cases in the red line. For n inputs, there are n! different orders of inputs. For n-bit binary messages, there are 2n output possibilities. As n increases, n! is much larger than 2n, which means that there must be some different input order that maps to the same binary message. This is why Alice can have many encryption options to choose from. When Bob gets the cipher text, he decrypts the cipher text through the DNA temporal logic circuit. The decrypted plain text message is decoded into plain text characters. The specific operation of Bob is shown in Figure 5c. It can be seen that when Bob decrypts the cipher text using the DNA temporal logic circuit, different input orders would generate different output results. Here, Bob only knows the order of the cipher text, not the specific content. When Bob uses I2, I3, I1, I5, and I4 as the input order, the decrypted binary message is (0,1,1,0,1). When Bob uses I3, I2, I5, I4, and I1 as the input order, the decrypted binary message is (0,0,1,0,1). When Bob uses I1, I2, I5, I4, and I3 as the input order, the decrypted binary message is (1,1,0,0,1). Finally, Bob decodes these binary messages and decrypts the corresponding plain text characters. It is worth noting that in the process of the actual reaction, the concentration change may cause errors in the decryption results. However, we tend to focus more on the logical values of 0 and 1 of the output than on the fluorescence intensity, so if we can properly delimit 0 and 1 (adjust the value of the specific separation between 0 and 1, or adjust the reaction time), errors due to concentration are considered acceptable in the decryption process. All fluorescence results for the decryption process were obtained using data from the simulation model, which shows that DNA temporal logic circuits can be used effectively for the symmetrically encrypted communication of binary messages.

## 4. Discussion

In this study, we proposed a general design rule for DNA temporal logic circuits, which could map temporally ordered inputs to different binary messages. We applied this scheme to symmetric encryption to protect binary information during communication. Accordingly, we demonstrated that our circuit had excellent responsiveness to temporally ordered inputs, flexibility, and expansibility, which could be applied to the symmetrically encrypted communication of binary messages.

It should be noted that the release and inhibition reactions were key in achieving the responses to different input orders, and the substrate could lead to different binary number outputs according to the different reactions that occurred. In the experiments, we set the temperature condition of these reactions to 37 °C; thus, our framework could adapt to the biological environment. Individual output signals could also be designed to participate in downstream reactions. All these features provided a modular interface for subsequent extensions and facilitate the potential implementation of more complex and larger-scale synthetic circuits. DNA circuits with a temporal memory function are considered to have the potential to provide more opportunities for the future intelligent behavior of artificial molecular machines [35]. We hope that our scheme provides some new ideas for future neural networks [36,37], information processing [12,38,39], and molecular encryption [40,41].

## Data Availability

Not applicable.

## Supplementary Materials

The following supporting information can be downloaded at: https://www.mdpi.com/article/10.3390/nano13050903/s1, Figure S1: Data of three inputs and two substrates temporal logic circuit; Figure S2: Data and simulation of three inputs and two substrates temporal logic circuit; Figure S3: Data of three inputs and three substrates temporal logic circuit; Figure S4: Data and simulation of three inputs and three substrates temporal logic circuit; Figure S5: Data of four inputs and three substrates temporal logic circuit; Figure S6: Data and simulation of four inputs and three substrates temporal logic circuit; Table S1: DNA sequences and modifications; Table S2: NUPACK simulations for DNA complexes; Table S3: Custom encoding and decoding of conversion between characters and binary message.
