# Supplementary material for: Mapping Temporally Ordered Inputs to Binary Message Outputs with a DNA Temporal Logic Circuit
Source: Nanomaterials (Basel). 2023 Feb 27;13(5):903. doi: 10.3390/nano13050903 (PMC10005157; doi:10.3390/nano13050903)
Supplement: Supplementary file 1 [file nanomaterials-13-00903-s001.zip › nanomaterials-2183229-supplementary.pdf]

nanomaterials

MDPI

Supplementary Materials

Mapping Temporally Ordered Inputs to Binary Message Outputs with a DNA Temporal Logic Circuit

Shuai Zhao [1,†], Yuan Liu [2,†], Xiaokang Zhang [2], Rui Qin [1], Bin Wang [1] and Qiang Zhang [1,*]

[1] Key Laboratory of Advanced Design and Intelligent Computing, Ministry of Education, School of Software Engineering, Dalian University, Dalian 116622, China; second6s9@163.com (S.Z.); qinrui9850@163.com (R.Q.); wangbin@dlu.edu.cn (B.W.); zhangq@dlu.edu.cn (Q.Z.)
[2] School of Computer Science and Technology, Dalian University of Technology, Dalian 116024, China; liuyuan.dlut@gmail.com (Y.L.); xiaokangzhangdl@gmail.com (X.Z.)
* Correspondence: zhangq@dlu.edu.cn; Tel.: +86-0411-8740-2106
† These authors contributed equally to this work.

S1. DNA Sequences Design and Modifications

We use the design module of NUPACK (www.nupack.org) for basic design of DNA sequences. Then manually modify the designed sequences to produce the details we need and use the analysis module of NUPACK to ensure that there are few undesirable interactions between DNA strands as possible.

Table S1. DNA sequences and modifications

Strand	Sequence(5' to 3')	Length (nt)
a1(O1)	6-FAM-GATTCTTGATACGACTCTTG	20
a2(O2)	ROX-GTCTCACTTGAACTCTGTCT	20
a3(O3)	CY5.5-CATCTTACATATCTTGATCT	20
b1(K1)	AACCATCTCACTGTCTGAAC	20
K2	TGGACAAACCATCTCACTGTCTGAAC	26
K3	CACTTCTGGACAAACCATCTCACTGTCTGAAC	32
I1*	GTTCAGACAGTGAGATGGTTTGTCCACAAGAGTCGTATCAAGAATC-BHQ1	46
I2*	GTTCAGACAGTGAGATGGTTTGTCCAGAAGTGAGACAGAGTTCAAGTGAGAC-BHQ2	52
I3*	GTTCAGACAGTGAGATGGTTTGTCCAGAAGTGACCAGAAGATCAAGATATGTAAGATG-BHQ3	58
I1	GATTCTTGATACGACTCTTGTGGACAAACCATCTCACTGTCTGAAC	46
I2	GTCTCACTTGAACTCTGTCTCACTTCTGGACAAACCATCTCACTGTCTGAAC	52
I3	CATCTTACATATCTTGATCTTCTGGTCACTTCTGGACAAACCATCTCACTGTCTGAAC	58
I4	CTGTTCTCTAACTTCATCAGGTAACTTCTGGTCACTTCTGGACAAACCATCTCACTGTCTGAAC	64

Domains of the DNA temporal logic circuit are colored as follows:

Domains a1 (O1) and a1*, domains a2 (O2) and a2*, domains a3 (O3) and a3*, domains a4, domains b1 (K1) and b1*.

All toehold domains are highlighted as follows:

Domains t1 and t1*, domains t2 and t2*, domains t3 and t3*, domains t4.

S2. NUPACK Simulations

We use NUPACK to analyze the stability of DNA complexes (table S2).

Table S2. NUPACK simulations for DNA complexes.

Complexes	MFE structure
S1	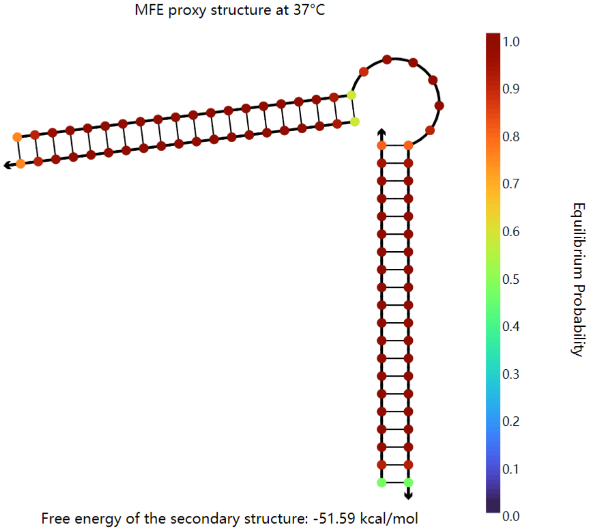
S2	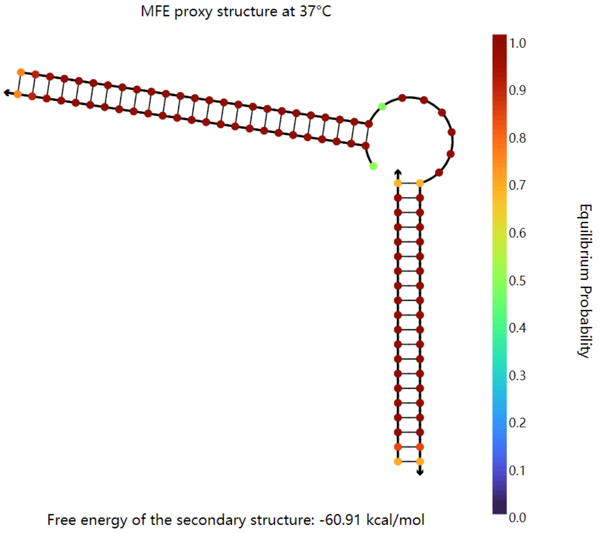
S3	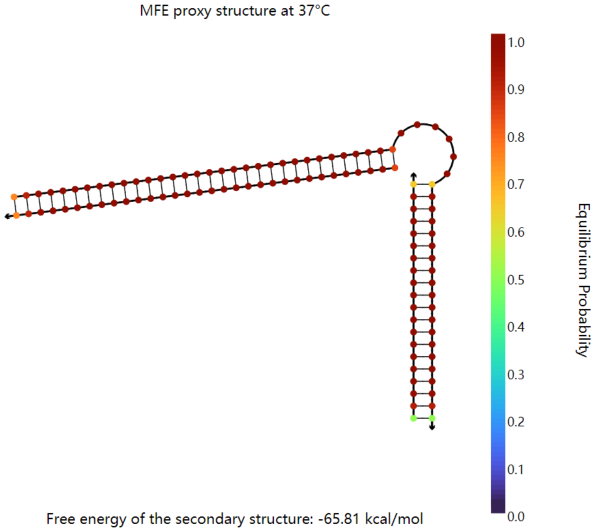

W11

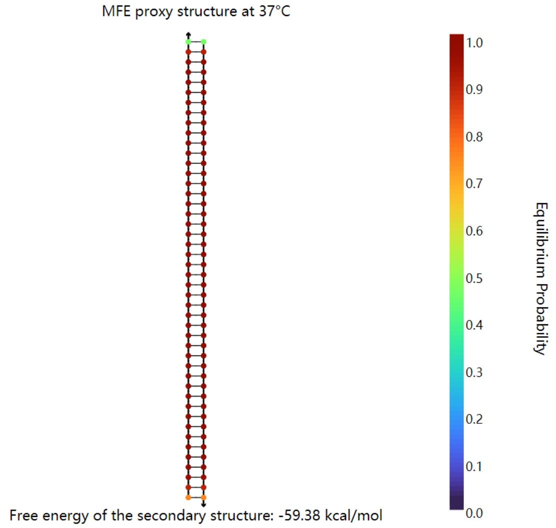
MFE proxy structure at 37°C
1.0
0.9
0.8
0.7
0.6
0.5
0.4
0.3
0.2
0.1
0.0
Equilibrium Probability
Free energy of the secondary structure: -59.38 kcal/mol

W22

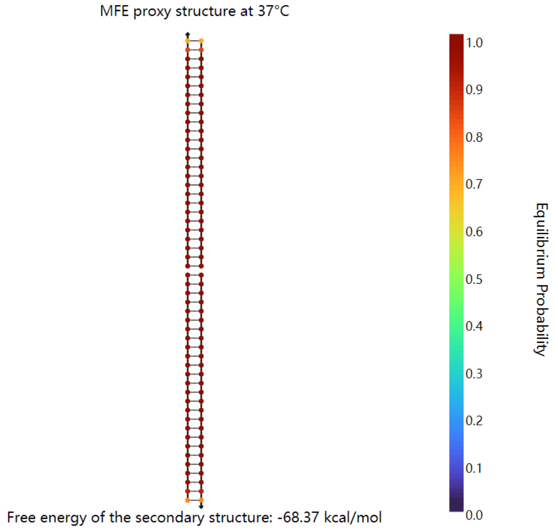
MFE proxy structure at 37°C
1.0
0.9
0.8
0.7
0.6
0.5
0.4
0.3
0.2
0.1
0.0
Equilibrium Probability
Free energy of the secondary structure: -68.37 kcal/mol

W33

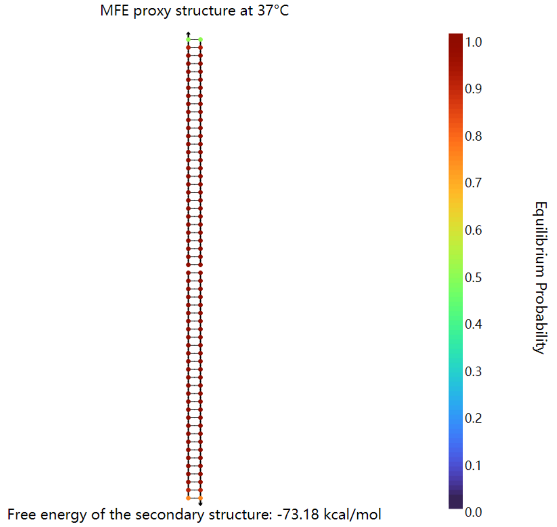
MFE proxy structure at 37°C
1.0
0.9
0.8
0.7
0.6
0.5
0.4
0.3
0.2
0.1
0.0
Equilibrium Probability
Free energy of the secondary structure: -73.18 kcal/mol

W21	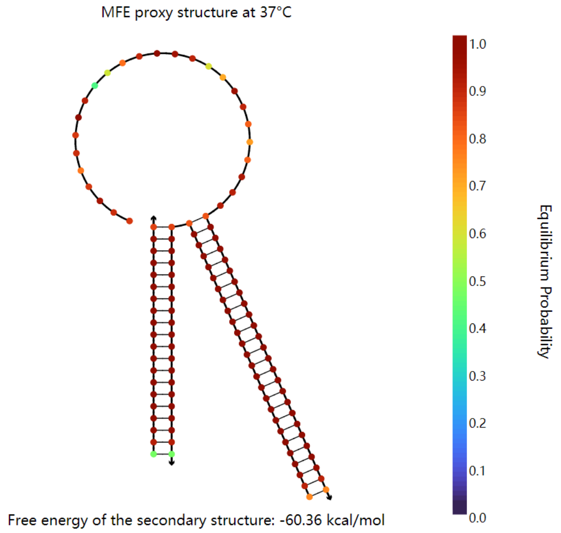 MFE proxy structure at 37°C<br>1.0<br>0.9<br>0.8<br>0.7<br>0.6<br>0.5<br>0.4<br>0.3<br>0.2<br>0.1<br>0.0<br>Equilibrium Probability<br>Free energy of the secondary structure: -60.36 kcal/mol
W31	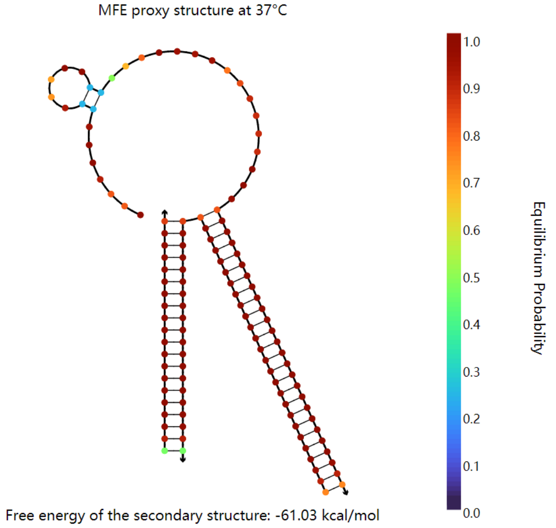 MFE proxy structure at 37°C<br>1.0<br>0.9<br>0.8<br>0.7<br>0.6<br>0.5<br>0.4<br>0.3<br>0.2<br>0.1<br>0.0<br>Equilibrium Probability<br>Free energy of the secondary structure: -61.03 kcal/mol
W32	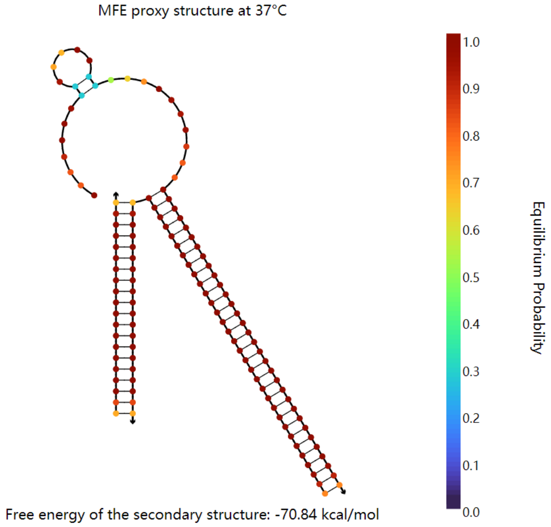 MFE proxy structure at 37°C<br>1.0<br>0.9<br>0.8<br>0.7<br>0.6<br>0.5<br>0.4<br>0.3<br>0.2<br>0.1<br>0.0<br>Equilibrium Probability<br>Free energy of the secondary structure: -70.84 kcal/mol

S3. Reaction Simulations Model

All release reactions were used to simulate output signal generation and modeled as follows:

$$I1 + S1 \xrightarrow{k1} O1 + B1 + W11 \quad (1)$$

$$I2 + S2 \xrightarrow{k2} O2 + B2 + W22 \quad (2)$$

$$I3 + S3 \xrightarrow{k3} O3 + B3 + W33 \quad (3)$$

$$I4 + S4 \xrightarrow{k4} O4 + B4 + W44 \quad (4)$$

$$I5 + S5 \xrightarrow{k5} O5 + B5 + W55 \quad (5)$$

All inhibition reactions were used to simulate the inhibition of output signal and modeled as follows:

$$I2 + S1 \xrightarrow{k6} B1 + W21 \quad (6)$$

$$I3 + S1 \xrightarrow{k7} B1 + W31 \quad (7)$$

$$I3 + S2 \xrightarrow{k8} B2 + W32 \quad (8)$$

$$I4 + S1 \xrightarrow{k9} B1 + W41 \quad (9)$$

$$I4 + S2 \xrightarrow{k10} B2 + W42 \quad (10)$$

$$I4 + S3 \xrightarrow{k11} B3 + W43 \quad (11)$$

$$I5 + S1 \xrightarrow{k12} B1 + W51 \quad (12)$$

$$I5 + S2 \xrightarrow{K13} B2 + W52 \quad (13)$$

$$I5 + S3 \xrightarrow{k14} B3 + W53 \quad (14)$$

$$I5 + S4 \xrightarrow{k15} B4 + W54 \quad (15)$$

The following reactions were used to simulate substrates loss:

$$B2 + S1 \xrightarrow{k6} B1 + WB21 \quad (16)$$

$$B3 + S1 \xrightarrow{k7} B1 + WB31 \quad (17)$$

$$B3 + S2 \xrightarrow{k8} B2 + WB32 \quad (18)$$

$$B4 + S1 \xrightarrow{k9} B1 + WB41 \quad (19)$$

$$B4 + S2 \xrightarrow{k10} B2 + WB42 \quad (20)$$

$$B4 + S3 \xrightarrow{k11} B3 + WB43 \quad (21)$$

$$B5 + S1 \xrightarrow{k12} B1 + WB51 \quad (22)$$

$$B5 + S2 \xrightarrow{k13} B2 + WB52 \quad (23)$$

$$B5 + S3 \xrightarrow{k14} B3 + WB53 \quad (24)$$

$$B5 + S4 \xrightarrow{k15} B4 + WB54 \quad (25)$$

Since the reactions of the same substrate have the same toehold domain and branch migration domain and approximate symmetry of the reactions, we assume the same rate

constant for the reactions of the same substrate. In other words, we assume that k1 = k6 = k7 = k9 = k12, k2 = k8= k10 = k13, k3 = k11 = k14, k4 = k15.

According to the above reaction model, we can find that there are two types of reaction. One is a reaction of two reactants and three products that can be abstracted into the following equation:

$$A + B \xrightarrow{ks1} C + D + E \tag{26}$$

The other is that the reaction of two reactants and two products can be abstracted into the following equation:

$$A + B \xrightarrow{ks2} C + D \tag{27}$$

Therefore, the rate equation of the first reaction type can be derived:

$$d[\mathrm{C}]/\mathrm{dt} = \mathrm{d[D]/dt} = \mathrm{d[E]/dt} = \mathrm{ks1[A][B]} \tag{28}$$

When the initial condition is $[A]_0$, $[B]_0$, the curve of products can be obtained either by integration or solving difference equations. For example, the differential equations above could be described by the following difference equations:

$$A(\mathrm{i+1}) = A(\mathrm{i}) - \mathrm{ks1\,A(i)\,B(i)} \tag{29}$$

$$\mathrm{B(i+1)} = B(\mathrm{i}) - \mathrm{ks1\,A(i)\,B(i)} \tag{30}$$

$$\mathrm{C(i+1)} = C(\mathrm{i}) + \mathrm{ks1\,A(i)\,B(i)} \tag{31}$$

$$\mathrm{D(i+1)} = D(\mathrm{i}) + \mathrm{ks1\,A(i)\,B(i)} \tag{32}$$

$$\mathrm{E(i+1)} = E(\mathrm{i}) + \mathrm{ks1\,A(i)\,B(i)} \tag{33}$$

Similarly, the reaction of the two products results in the following difference equation:

$$\mathrm{A(i+1)} = A(\mathrm{i}) - \mathrm{ks\,2\,A(i)\,B(i)} \tag{34}$$

$$\mathrm{B(i+1)} = B(\mathrm{i}) - \mathrm{ks\,2\,A(i)\,B(i)} \tag{35}$$

$$\mathrm{C(i+1)} = C(\mathrm{i}) + \mathrm{ks\,2\,A(i)\,B(i)} \tag{36}$$

$$\mathrm{D(i+1)} = D(\mathrm{i}) + \mathrm{ks\,2\,A(i)\,B(i)} \tag{37}$$

We use a python program to record the concentrations of reactants and products at each instant and create models based on reaction relationships. In the program, we set the initial concentration of all substrates. When an input is added, we assign an initial concentration to that input in the program and compute the difference equation for each reaction.

For the reaction model with three inputs and two substrates, all possible reactions that can occur are (1) (2) (6) (7) (8) (16) (17) (18).

For the reaction model with three inputs and three substrates, all possible reactions that can occur are (1) (2) (3) (6) (7) (8) (16) (17) (18).

For the reaction model with four inputs and three substrates, all possible reactions that can occur are (1) (2) (3) (6) (7) (8) (9) (10) (11) (16) (17) (18) (19) (20) (21).

For the reaction model with five inputs and five substrates, all possible reactions that can occur are (1) - (25). In this simulation model, the substrate loss was 0 because no actual experiments were performed. Therefore, the highest fluorescence results △F / △Fmax of all experimental results were 1.

S4. Supplementary Data and Simulation

S4.1. Temporal logic circuits with three inputs and two substrates

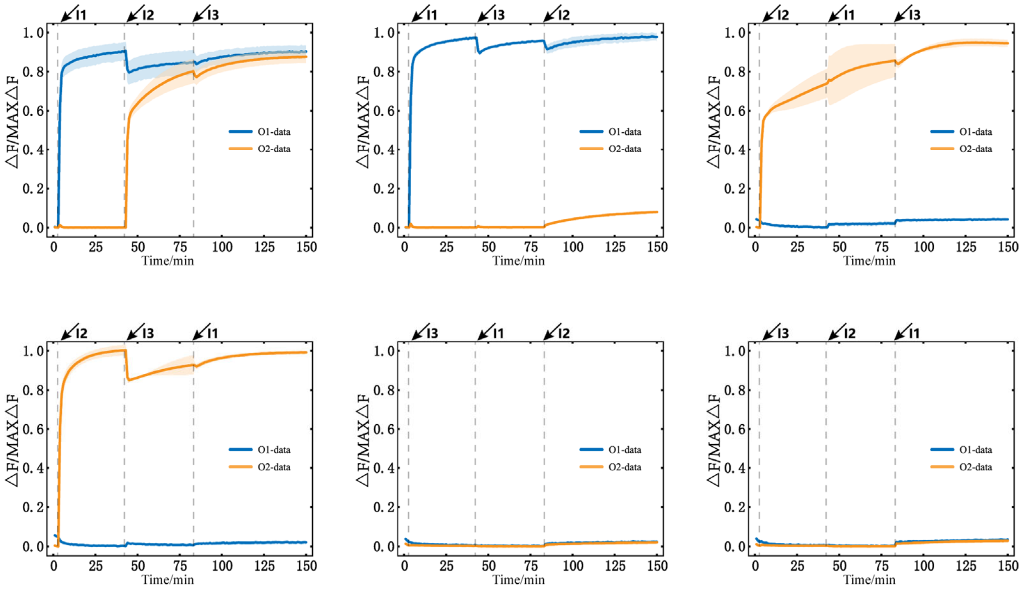

Figure S1. Data of three inputs and two substrates temporal logic circuit. All resulting plots contain error bars. We examined the experiment for all six orders of three inputs and plotted the fluorescence curves.

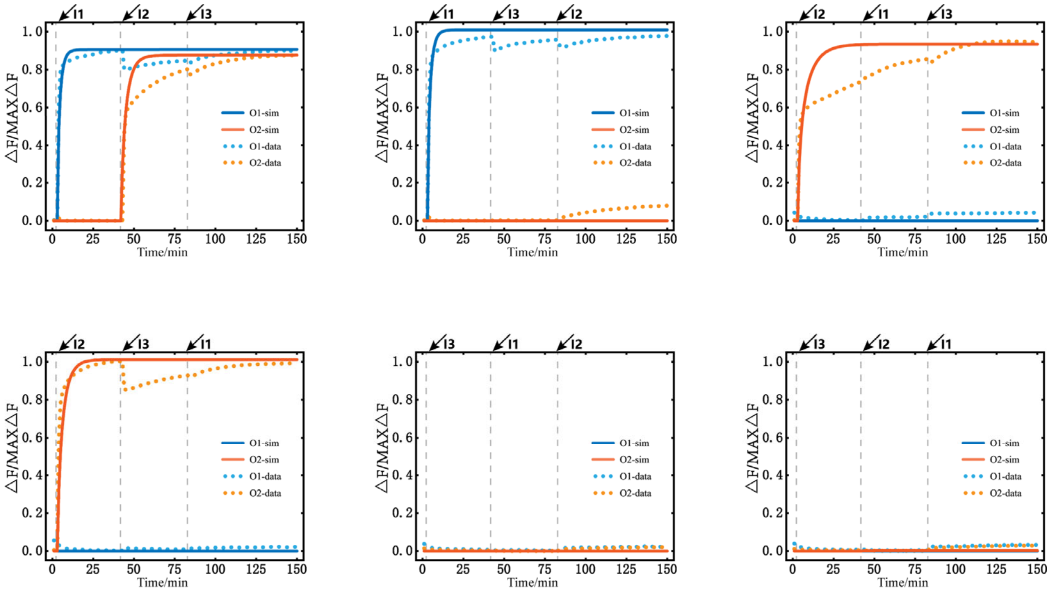

Figure S2. Data and simulation of three inputs and two substrates temporal logic circuit. The dashed line represents the real data and the solid line represents the simulation curve. The figure contains the results for all six orders for the three inputs.

S4.2. Temporal logic circuits with three inputs and three substrates

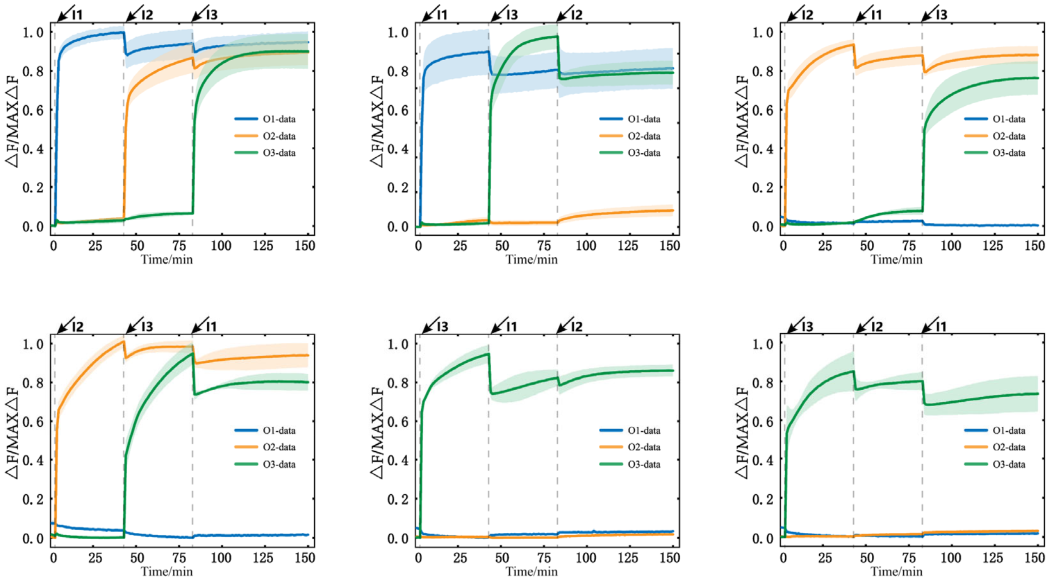

Figure S3. Data of three inputs and three substrates temporal logic circuit. All resulting plots contain error bars. We examined the experiment for all six orders of three inputs and plotted the fluorescence curves.

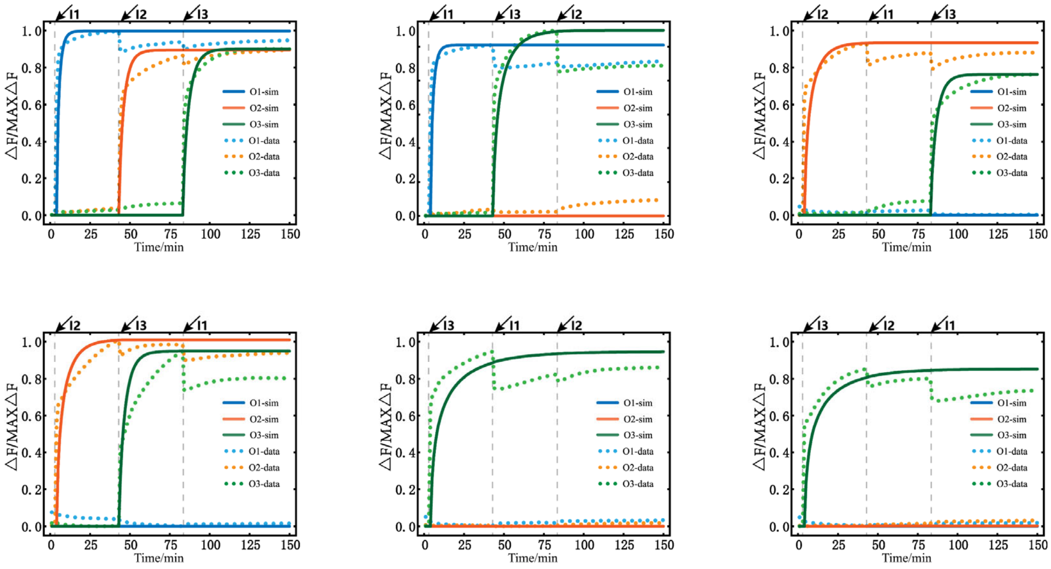

Figure S4. Data and simulation of three inputs and three substrates temporal logic circuit. The dashed line represents the real data and the solid line represents the simulation curve. The figure contains the results for all six orders for the three inputs.

S4.3. Temporal logic circuits with four inputs and three substrates

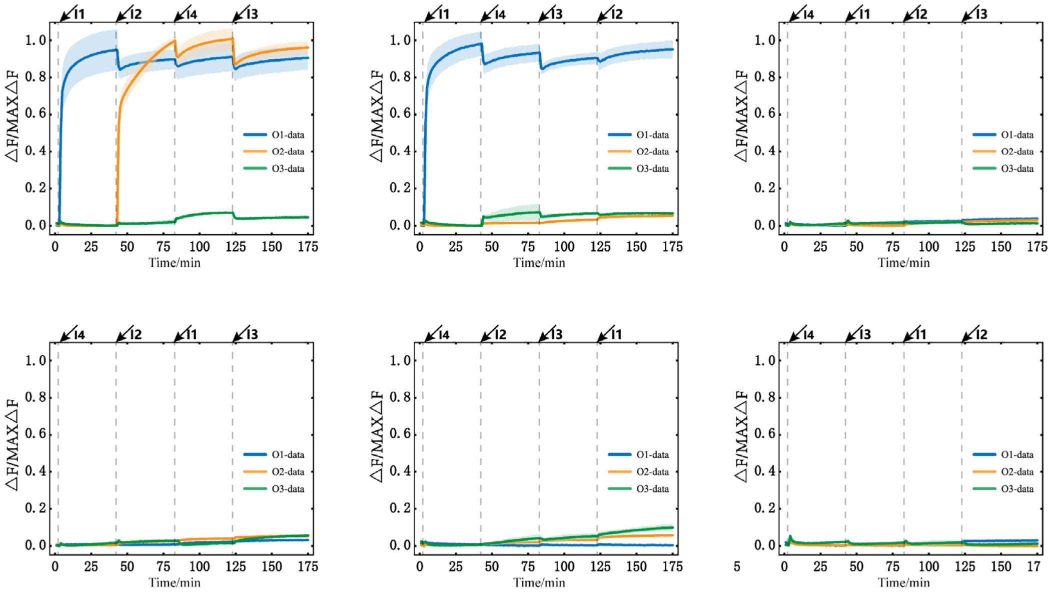

Figure S5. Data of four inputs and three substrates temporal logic circuit. Since we only examine the inhibitory effect of I4 here, the experiments were performed for six input orders only and suffice to show that the addition of I4 inhibits substrates S1, S2 and S3.

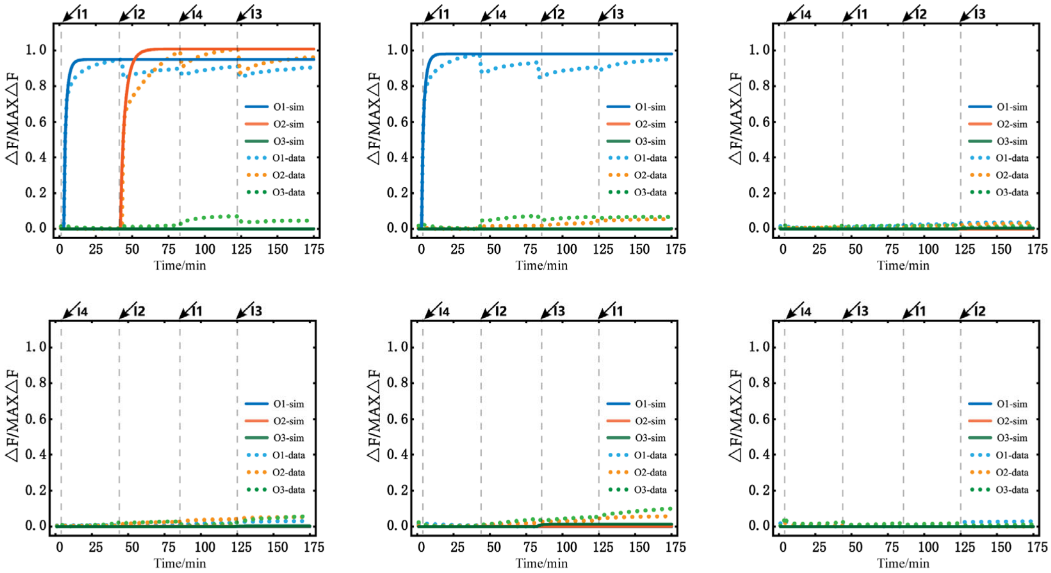

Figure S6. Data and simulation of four inputs and three substrates temporal logic circuit. The dashed line represents the real data and the solid line represents the simulation curve.

S5. Encoding and Decoding of Symmetric Encryption Applications

Table S3. Custom encoding and decoding of conversion between characters and binary message.

	Characters	Binary Message (plaintext message)
1	A	00001
2	B	00010
3	C	00011
4	D	00100
5	E	00101

6	F	00110
7	G	00111
8	H	01000
9	I	01001
10	J	01010
11	K	01011
12	L	01100
13	M	01101
14	N	01110
15	O	01111
16	P	10000
17	Q	10001
18	R	10010
19	S	10011
20	T	10100
21	U	10101
22	V	10110
23	W	10111
24	X	11000
25	Y	11001
26	Z	11010

S6. The Python Program Used by the Simulations Model

All programs are run in python3.8.5. The code for each simulation model is as follows.

S6.1. Three inputs and two substrates

```
import matplotlib.pyplot as plt
import numpy
import openpyxl

plt.figure(figsize=(10, 8))
timeMM = 1
dataNum = 150
p = 0

real_xi = numpy.linspace(0, 0, dataNum)
real_fam = numpy.linspace(0, 0, dataNum)
real_rox = numpy.linspace(0, 0, dataNum)
real_cy5_5 = numpy.linspace(0, 0, dataNum)
wb = openpyxl.load_workbook('double/Order123.xlsx')
# wb = openpyxl.load_workbook('double /Order132.xlsx')
# wb = openpyxl.load_workbook('double /Order213.xlsx')
# wb = openpyxl.load_workbook('double /Order231.xlsx')
# wb = openpyxl.load_workbook('double /Order312.xlsx')
# wb = openpyxl.load_workbook('double /Order321.xlsx')
sheet = wb['Sheet1']
c_fam = 0
c_rox = 0
c_cy5_5 = 0
for i in range(0, dataNum):
    real_xi[i] = i
    real_fam[i] = sheet.cell(row=2 + i, column=1).value
    real_rox[i] = sheet.cell(row=2 + i, column=2).value
    real_cy5_5[i] = sheet.cell(row=2 + i, column=3).value
```

```
    c_fam = max(c_fam, real_fam[i])
    c_rox = max(c_rox, real_rox[i])
    c_cy5_5 = max(c_cy5_5, real_cy5_5[i])

k1 = 0.00150
k2 = 0.00090
k3 = 0.00075

I1 = numpy.linspace(0, 0, dataNum)
I2 = numpy.linspace(0, 0, dataNum)
I3 = numpy.linspace(0, 0, dataNum)

S1 = numpy.linspace(0, 0, dataNum)
S2 = numpy.linspace(0, 0, dataNum)
S3 = numpy.linspace(0, 0, dataNum)

O1 = numpy.linspace(0, 0, dataNum)
O2 = numpy.linspace(0, 0, dataNum)
O3 = numpy.linspace(0, 0, dataNum)

B1 = numpy.linspace(0, 0, dataNum)
B2 = numpy.linspace(0, 0, dataNum)
B3 = numpy.linspace(0, 0, dataNum)

W11 = numpy.linspace(0, 0, dataNum)
W22 = numpy.linspace(0, 0, dataNum)
W33 = numpy.linspace(0, 0, dataNum)
W21 = numpy.linspace(0, 0, dataNum)
W31 = numpy.linspace(0, 0, dataNum)
W32 = numpy.linspace(0, 0, dataNum)
WB21 = numpy.linspace(0, 0, dataNum)
WB31 = numpy.linspace(0, 0, dataNum)
WB32 = numpy.linspace(0, 0, dataNum)

l = [I1, I2, I3, S1, S2, S3, O1, O2, O3, B1, B2, B3, W11, W22, W33, W21, W31, W32, WB21, WB31, WB32]
xi = numpy.linspace(0, 0, dataNum)

n = 3

def initial():
    S1[0] = c_fam
    S2[0] = c_rox
    for i in range(0, dataNum):
        xi[i] = i
def reaction1(A, B, C, D, E, k, i):
    # A + B -> C + D + E
    if i + 1 < dataNum:
        det = k * A[i] * B[i]
        A[i + 1] = (A[i] - det) if A[i + 1] == 0 else (A[i + 1] - det)
        B[i + 1] = (B[i] - det) if B[i + 1] == 0 else (B[i + 1] - det)
        C[i + 1] = (C[i] + det) if C[i + 1] == 0 else (C[i + 1] + det)
        D[i + 1] = (D[i] + det) if D[i + 1] == 0 else (D[i + 1] + det)
        E[i + 1] = (E[i] + det) if E[i + 1] == 0 else (E[i + 1] + det)
```

```
def reaction2(A, B, C, D, k, i):
    # A + B -> C + D
    if i + 1 < dataNum:
        det = k * A[i] * B[i]
        A[i + 1] = (A[i] - det) if A[i + 1] == 0 else (A[i + 1] - det)
        B[i + 1] = (B[i] - det) if B[i + 1] == 0 else (B[i + 1] - det)
        C[i + 1] = (C[i] + det) if C[i + 1] == 0 else (C[i + 1] + det)
        D[i + 1] = (D[i] + det) if D[i + 1] == 0 else (D[i + 1] + det)

def reaction_parallel(Input, start, end):
    if start != 0:
        start -= 1
    Input[start] = n * 120
    for i in range(start, end):
        reaction1(I1, S1, O1, B1, W11, k1, i)
        reaction1(I2, S2, O2, B2, W22, k2, i)
        reaction2(I2, S1, B1, W21, k1, i)
        reaction2(I3, S1, B1, W31, k1, i)
        reaction2(I3, S2, B2, W32, k2, i)
        reaction2(B2, S1, B1, WB21, k1, i)
        reaction2(B3, S1, B1, WB31, k1, i)
        reaction2(B3, S2, B2, WB32, k2, i)

def reaction_orders(Input1, Input2, Input3, time):
    start = 0
    end = time
reaction_parallel(Input1, start, end)

    start = time
    end = 2 * time
    reaction_parallel(Input2, start, end)

    start = 2 * time
    end = dataNum
    reaction_parallel(Input3, start, end)

def addInitial(xi, dataNum):
    ti = numpy.linspace(0, 0, dataNum)
    ti[0] = 0
    ti[1] = 0
    ti[2] = 0
    for i in range(0, dataNum):
        if i + 3 < dataNum:
            ti[i + 3] = abs(xi[i])

    return ti

initial()
reaction_orders(I1, I2, I3, 40)
# reaction_orders(I1,I3,I2,40)
# reaction_orders(I2, I1, I3, 40)
# reaction_orders(I2, I3, I1, 40)
# reaction_orders(I3, I1, I2, 40)
```

```
# reaction_orders(I3, I2, I1, 40)

O1 = addInitial(O1, dataNum)
O2 = addInitial(O2, dataNum)
O3 = addInitial(O3, dataNum)

ax=plt.gca()
ax.spines['top'].set_linewidth(2)
ax.spines['bottom'].set_linewidth(2)
ax.spines['left'].set_linewidth(2)
ax.spines['right'].set_linewidth(2)

plt.xlabel('Time(Min)', fontsize=20)
plt.ylabel('Fluorescence(n.u.)', fontsize=20)

plt.plot(real_xi,real_fam,color='#9BC2E6',linewidth=2.0,linestyle='--')
plt.plot(real_xi,real_rox,color='#F4B084',linewidth=2.0,linestyle='--')

plt.plot(xi, O1, color="#0070C0", label="O1", linewidth=6)
plt.plot(xi, O2, color="#ED7D31", label="O2", linewidth=6)

plt.tick_params(labelsize=20)
plt.legend(fontsize=20)
plt.show()
```

S6.2. Three inputs and three substrates

```
import matplotlib.pyplot as plt
import numpy
import openpyxl

plt.figure(figsize=(10, 8))
timeMM = 1
dataNum = 150
p = 0

real_xi = numpy.linspace(0, 0, dataNum)
real_fam = numpy.linspace(0, 0, dataNum)
real_rox = numpy.linspace(0, 0, dataNum)
real_cy5_5 = numpy.linspace(0, 0, dataNum)
# wb = openpyxl.load_workbook('triple/Order123.xlsx')
# wb = openpyxl.load_workbook('triple/Order132.xlsx')
# wb = openpyxl.load_workbook('triple/Order213.xlsx')
# wb = openpyxl.load_workbook('triple/Order231.xlsx')
# wb = openpyxl.load_workbook('triple/Order312.xlsx')
wb = openpyxl.load_workbook('triple/Order321.xlsx')
sheet = wb['Sheet1']
c_fam = 0
c_rox = 0
c_cy5_5 = 0
for i in range(0, dataNum):
    real_xi[i] = i
    real_fam[i] = sheet.cell(row=2 + i, column=1).value
    real_rox[i] = sheet.cell(row=2 + i, column=2).value
    real_cy5_5[i] = sheet.cell(row=2 + i, column=3).value
```

```
        c_fam = max(c_fam, real_fam[i])
        c_rox = max(c_rox, real_rox[i])
        c_cy5_5 = max(c_cy5_5, real_cy5_5[i])

    if c_fam < 40:
        c_fam = 120

    if c_rox < 40:
        c_rox = 120

    if c_cy5_5 < 40:
        c_cy5_5 = 120

    k1 = 0.00150
    k2 = 0.00090
    k3 = 0.00075

    I1 = numpy.linspace(0, 0, dataNum)
    I2 = numpy.linspace(0, 0, dataNum)
    I3 = numpy.linspace(0, 0, dataNum)

    S1 = numpy.linspace(0, 0, dataNum)
    S2 = numpy.linspace(0, 0, dataNum)
    S3 = numpy.linspace(0, 0, dataNum)

    O1 = numpy.linspace(0, 0, dataNum)
    O2 = numpy.linspace(0, 0, dataNum)
    O3 = numpy.linspace(0, 0, dataNum)

    B1 = numpy.linspace(0, 0, dataNum)
    B2 = numpy.linspace(0, 0, dataNum)
    B3 = numpy.linspace(0, 0, dataNum)

    W11 = numpy.linspace(0, 0, dataNum)
    W22 = numpy.linspace(0, 0, dataNum)
    W33 = numpy.linspace(0, 0, dataNum)
    W21 = numpy.linspace(0, 0, dataNum)
    W31 = numpy.linspace(0, 0, dataNum)
    W32 = numpy.linspace(0, 0, dataNum)
    WB21 = numpy.linspace(0, 0, dataNum)
    WB31 = numpy.linspace(0, 0, dataNum)
    WB32 = numpy.linspace(0, 0, dataNum)

    l = [I1, I2, I3, S1, S2, S3, O1, O2, O3, B1, B2, B3, W11, W22, W33, W21, W31, W32, WB21, WB31, WB32]
    xi = numpy.linspace(0, 0, dataNum)
    n = 3

    def initial():

        S1[0] = c_fam
        S2[0] = c_rox
        S3[0] = c_cy5_5
        for i in range(0, dataNum):
```

```
        xi[i] = i

def reaction1(A, B, C, D, E, k, i):
    # A + B -> C + D + E
    if i + 1 < dataNum:
        det = k * A[i] * B[i]
        A[i + 1] = (A[i] - det) if A[i + 1] == 0 else (A[i + 1] - det)
        B[i + 1] = (B[i] - det) if B[i + 1] == 0 else (B[i + 1] - det)
        C[i + 1] = (C[i] + det) if C[i + 1] == 0 else (C[i + 1] + det)
        D[i + 1] = (D[i] + det) if D[i + 1] == 0 else (D[i + 1] + det)
        E[i + 1] = (E[i] + det) if E[i + 1] == 0 else (E[i + 1] + det)

def reaction2(A, B, C, D, k, i):
    # A + B -> C + D
    if i + 1 < dataNum:
        det = k * A[i] * B[i]
        A[i + 1] = (A[i] - det) if A[i + 1] == 0 else (A[i + 1] - det)
        B[i + 1] = (B[i] - det) if B[i + 1] == 0 else (B[i + 1] - det)
        C[i + 1] = (C[i] + det) if C[i + 1] == 0 else (C[i + 1] + det)
        D[i + 1] = (D[i] + det) if D[i + 1] == 0 else (D[i + 1] + det)

def reaction_parallel(Input, start, end):
    if start != 0:
        start -= 1
    Input[start] = n * 120
    for i in range(start, end):
        reaction1(I1, S1, O1, B1, W11, k1, i)
        reaction1(I2, S2, O2, B2, W22, k2, i)
        reaction1(I3, S3, O3, B3, W33, k3, i)

        reaction2(I2, S1, B1, W21, k1, i)
        reaction2(I3, S1, B1, W31, k1, i)
        reaction2(I3, S2, B2, W32, k2, i)

        reaction2(B2, S1, B1, WB21, k1, i)
        reaction2(B3, S1, B1, WB31, k1, i)
        reaction2(B3, S2, B2, WB32, k2, i)

def reaction_orders(Input1, Input2, Input3, time):
    # plt.title(Input1 + '-' + Input2 + '-' + Input3, fontsize=40)
    start = 0
    end = time
    reaction_parallel(Input1, start, end)

    start = time
    end = 2 * time
    reaction_parallel(Input2, start, end)

    start = 2 * time
    end = dataNum
    reaction_parallel(Input3, start, end)

def addInitial(xi, dataNum):
    ti = numpy.linspace(0, 0, dataNum)
```

```
    ti[0] = 0
    ti[1] = 0
    ti[2] = 0
    for i in range(0, dataNum):
        if i + 3 < dataNum:
            ti[i + 3] = abs(xi[i])

    return ti

initial()
# reaction_orders(I1, I2, I3, 40)
# reaction_orders(I1,I3,I2,40)
# reaction_orders(I2, I1, I3, 40)
# reaction_orders(I2, I3, I1, 40)
# reaction_orders(I3, I1, I2, 40)
reaction_orders(I3, I2, I1, 40)

O1 = addInitial(O1, dataNum)
O2 = addInitial(O2, dataNum)
O3 = addInitial(O3, dataNum)

ax=plt.gca()
ax.spines['top'].set_linewidth(2)
ax.spines['bottom'].set_linewidth(2)
ax.spines['left'].set_linewidth(2)
ax.spines['right'].set_linewidth(2)

plt.xlabel('Time(Min)', fontsize=20)
plt.ylabel('Fluorescence(n.u.)', fontsize=20)

plt.plot(real_xi,real_fam,color='#9BC2E6',linewidth=2.0,linestyle='--')
plt.plot(real_xi,real_rox,color='#F4B084',linewidth=2.0,linestyle='--')
plt.plot(real_xi,real_cy5_5,color='#A9D08E',linewidth=2.0,linestyle='--')

plt.plot(xi, O1, color="#0070C0", label="O1", linewidth=6)
plt.plot(xi, O2, color="#ED7D31", label="O2", linewidth=6)
plt.plot(xi, O3, color="#548235", label="O3", linewidth=6)

plt.tick_params(labelsize=20)
plt.legend(fontsize=20)
plt.show()
```

S6.3. Four inputs and three substrates

```
import matplotlib.pyplot as plt
import numpy
import openpyxl

plt.figure(figsize=(10, 8))
timeMM = 1
dataNum = 175
p = 0

k1 = 0.00150
k2 = 0.00090
```

```
k3 = 0.00075
real_xi = numpy.linspace(0, 0, dataNum)
real_fam = numpy.linspace(0, 0, dataNum)
real_rox = numpy.linspace(0, 0, dataNum)
real_cy5_5 = numpy.linspace(0, 0, dataNum)

c_fam = 0
c_rox = 0
c_cy5_5 = 0

# wb = openpyxl.load_workbook('fourth/Order1243.xlsx')
# wb = openpyxl.load_workbook('fourth/Order1423.xlsx')
# wb = openpyxl.load_workbook('fourth/Order3412.xlsx')
# wb = openpyxl.load_workbook('fourth/Order4123.xlsx')
# wb = openpyxl.load_workbook('fourth/Order4213.xlsx')
# wb = openpyxl.load_workbook('fourth/Order4231.xlsx')
wb = openpyxl.load_workbook('fourth/Order4312.xlsx')
# wb = openpyxl.load_workbook('fourth/Order4321.xlsx')
sheet = wb['Sheet1']
for i in range(0, dataNum):
    real_xi[i] = i
    real_fam[i] = sheet.cell(row=2 + i, column=1).value
    real_rox[i] = sheet.cell(row=2 + i, column=2).value
    real_cy5_5[i] = sheet.cell(row=2 + i, column=3).value
    c_fam = max(c_fam, real_fam[i])
    c_rox = max(c_rox, real_rox[i])
    c_cy5_5 = max(c_cy5_5, real_cy5_5[i])

I1 = numpy.linspace(0, 0, dataNum)
I2 = numpy.linspace(0, 0, dataNum)
I3 = numpy.linspace(0, 0, dataNum)
I4 = numpy.linspace(0, 0, dataNum)
inputs = [I1, I2, I3, I4]
inputs_str = ["I1", "I2", "I3", "I4"]

S1 = numpy.linspace(0, 0, dataNum)
S2 = numpy.linspace(0, 0, dataNum)
S3 = numpy.linspace(0, 0, dataNum)

O1 = numpy.linspace(0, 0, dataNum)
O2 = numpy.linspace(0, 0, dataNum)
O3 = numpy.linspace(0, 0, dataNum)

B1 = numpy.linspace(0, 0, dataNum)
B2 = numpy.linspace(0, 0, dataNum)
B3 = numpy.linspace(0, 0, dataNum)

W11 = numpy.linspace(0, 0, dataNum)
W22 = numpy.linspace(0, 0, dataNum)
W33 = numpy.linspace(0, 0, dataNum)
W21 = numpy.linspace(0, 0, dataNum)
W31 = numpy.linspace(0, 0, dataNum)
W32 = numpy.linspace(0, 0, dataNum)
W41 = numpy.linspace(0, 0, dataNum)
```

```
W42 = numpy.linspace(0, 0, dataNum)
W43 = numpy.linspace(0, 0, dataNum)
WB21 = numpy.linspace(0, 0, dataNum)
WB31 = numpy.linspace(0, 0, dataNum)
WB32 = numpy.linspace(0, 0, dataNum)

l = [I1, I2, I3, I4, S1, S2, S3, O1, O2, O3, B1, B2, B3, W11, W22, W33, W21, W31, W32, W41, W42, W43, WB21, WB31, WB32]
xi = numpy.linspace(0, 0, dataNum)

n = 3

def initial():
    for obj in l:
        for i in range(dataNum):
            obj[i] = 0

    S1[0] = c_fam
    S2[0] = c_rox
    S3[0] = c_cy5_5
    for i in range(0, dataNum):
        xi[i] = i

def reaction1(A, B, C, D, E, k, i):
    # A + B -> C + D + E
    if i + 1 < dataNum:
        det = k * A[i] * B[i]
        A[i + 1] = (A[i] - det) if A[i + 1] == 0 else (A[i + 1] - det)
        B[i + 1] = (B[i] - det) if B[i + 1] == 0 else (B[i + 1] - det)
        C[i + 1] = (C[i] + det) if C[i + 1] == 0 else (C[i + 1] + det)
        D[i + 1] = (D[i] + det) if D[i + 1] == 0 else (D[i + 1] + det)
        E[i + 1] = (E[i] + det) if E[i + 1] == 0 else (E[i + 1] + det)

def reaction2(A, B, C, D, k, i):
    # A + B -> C + D
    if i + 1 < dataNum:
        det = k * A[i] * B[i]
        A[i + 1] = (A[i] - det) if A[i + 1] == 0 else (A[i + 1] - det)
        B[i + 1] = (B[i] - det) if B[i + 1] == 0 else (B[i + 1] - det)
        C[i + 1] = (C[i] + det) if C[i + 1] == 0 else (C[i + 1] + det)
        D[i + 1] = (D[i] + det) if D[i + 1] == 0 else (D[i + 1] + det)

def reaction_parallel(Input, start, end):
    if start != 0:
        start -= 1
    Input[start] = n * 120
    for i in range(start, end):
        # Release Reaction
        reaction1(I1, S1, O1, B1, W11, k1, i)
        reaction1(I2, S2, O2, B2, W22, k2, i)
        reaction1(I3, S3, O3, B3, W33, k3, i)

        # Inhibite Reaction
        reaction2(I2, S1, B1, W21, k1, i)
```

```
        reaction2(I3, S1, B1, W31, k1, i)
        reaction2(I3, S2, B2, W32, k2, i)
        reaction2(I4, S1, B1, W41, k1, i)
        reaction2(I4, S2, B2, W42, k2, i)
        reaction2(I4, S3, B3, W43, k3, i)

        # Kill Reaction
        reaction2(B2, S1, B1, WB21, k1, i)
        reaction2(B3, S1, B1, WB31, k1, i)
        reaction2(B3, S2, B2, WB32, k2, i)

def reaction_orders(Input1, Input2, Input3, Input4, time, isOpen = False):
    initial()
    start = 0
    end = time
    reaction_parallel(Input1, start, end)

    start = time
    end = 2 * time
    reaction_parallel(Input2, start, end)

    start = 2 * time
    end = 3 * time
    reaction_parallel(Input3, start, end)

    start = 3 * time
    end = dataNum
    reaction_parallel(Input4, start, end)

    if isOpen:
        openall(xi)

def show():
    ax=plt.gca()
    ax.spines['top'].set_linewidth(2)
    ax.spines['bottom'].set_linewidth(2)
    ax.spines['left'].set_linewidth(2)
    ax.spines['right'].set_linewidth(2)

    plt.xlabel('Time(S)', fontsize=20)
    plt.ylabel('Concentration(NM)', fontsize=20)

    plt.plot(xi, O1, color="green", label="O1", linewidth=6)
    plt.plot(xi, O2, color="orange", label="O2", linewidth=6)
    plt.plot(xi, O3, color="blue", label="O3", linewidth=6)
    plt.tick_params(labelsize=20)
    plt.legend(fontsize=20)
    plt.show()

def addInitial(xi, dataNum):
    ti = numpy.linspace(0, 0, dataNum)
    ti[0] = 0
    ti[1] = 0
    ti[2] = 0
```

```
    for i in range(0, dataNum):
        if i + 3 < dataNum:
            ti[i + 3] = abs(xi[i])

return ti

flag = False
# reaction_orders(I1,I2,I4,I3,40,flag)
# reaction_orders(I1,I4,I2,I3,40,flag)
# reaction_orders(I3,I4,I1,I2,40,flag)
# reaction_orders(I4,I1,I2,I3,40,flag)
# reaction_orders(I4,I2,I1,I3,40,flag)
# reaction_orders(I4,I2,I3,I1,40,flag)
reaction_orders(I4,I3,I1,I2,40,flag)
# reaction_orders(I4,I3,I2,I1,40,flag)
O1 = addInitial(O1, dataNum)
O2 = addInitial(O2, dataNum)
O3 = addInitial(O3, dataNum)

ax=plt.gca()
ax.spines['top'].set_linewidth(2)
ax.spines['bottom'].set_linewidth(2)
ax.spines['left'].set_linewidth(2)
ax.spines['right'].set_linewidth(2)

plt.xlabel('Time(Min)', fontsize=20)
plt.ylabel('Fluorescence(n.u.)', fontsize=20)

plt.plot(real_xi,real_fam,color='#9BC2E6',linewidth=2.0,linestyle='--')
plt.plot(real_xi,real_rox,color='#F4B084',linewidth=2.0,linestyle='--')
plt.plot(real_xi,real_cy5_5,color='#A9D08E',linewidth=2.0,linestyle='--')

plt.plot(xi, O1, color="#0070C0", label="O1", linewidth=6)
plt.plot(xi, O2, color="#ED7D31", label="O2", linewidth=6)
plt.plot(xi, O3, color="#548235", label="O3", linewidth=6)

plt.tick_params(labelsize=20)
plt.legend(fontsize=20)
plt.show()
```

S6.4. Five inputs and five substrates

```
import matplotlib.pyplot as plt
import numpy

plt.figure(figsize=(10, 8))
timeMM = 1
dataNum = 200
p = 0

k1 = 0.00150
k2 = 0.00090
k3 = 0.00075
k4 = 0.00070
k5 = 0.00065
```

```
c = 100
n = 5

I1 = numpy.linspace(0, 0, dataNum)
I2 = numpy.linspace(0, 0, dataNum)
I3 = numpy.linspace(0, 0, dataNum)
I4 = numpy.linspace(0, 0, dataNum)
I5 = numpy.linspace(0, 0, dataNum)
inputs = [I1, I2, I3, I4, I5]
inputs_str = ["I1", "I2", "I3", "I4", "I5"]

S1 = numpy.linspace(0, 0, dataNum)
S2 = numpy.linspace(0, 0, dataNum)
S3 = numpy.linspace(0, 0, dataNum)
S4 = numpy.linspace(0, 0, dataNum)
S5 = numpy.linspace(0, 0, dataNum)

O1 = numpy.linspace(0, 0, dataNum)
O2 = numpy.linspace(0, 0, dataNum)
O3 = numpy.linspace(0, 0, dataNum)
O4 = numpy.linspace(0, 0, dataNum)
O5 = numpy.linspace(0, 0, dataNum)

B1 = numpy.linspace(0, 0, dataNum)
B2 = numpy.linspace(0, 0, dataNum)
B3 = numpy.linspace(0, 0, dataNum)
B4 = numpy.linspace(0, 0, dataNum)
B5 = numpy.linspace(0, 0, dataNum)

W11 = numpy.linspace(0, 0, dataNum)
W22 = numpy.linspace(0, 0, dataNum)
W33 = numpy.linspace(0, 0, dataNum)
W44 = numpy.linspace(0, 0, dataNum)
W55 = numpy.linspace(0, 0, dataNum)

W21 = numpy.linspace(0, 0, dataNum)
W31 = numpy.linspace(0, 0, dataNum)
W32 = numpy.linspace(0, 0, dataNum)
W41 = numpy.linspace(0, 0, dataNum)
W42 = numpy.linspace(0, 0, dataNum)
W43 = numpy.linspace(0, 0, dataNum)
W51 = numpy.linspace(0, 0, dataNum)
W52 = numpy.linspace(0, 0, dataNum)
W53 = numpy.linspace(0, 0, dataNum)
W54 = numpy.linspace(0, 0, dataNum)
WB21 = numpy.linspace(0, 0, dataNum)
WB31 = numpy.linspace(0, 0, dataNum)
WB32 = numpy.linspace(0, 0, dataNum)
WB43 = numpy.linspace(0, 0, dataNum)
WB42 = numpy.linspace(0, 0, dataNum)
WB41 = numpy.linspace(0, 0, dataNum)
WB51 = numpy.linspace(0, 0, dataNum)
WB52 = numpy.linspace(0, 0, dataNum)
```

```
WB53 = numpy.linspace(0, 0, dataNum)
WB54 = numpy.linspace(0, 0, dataNum)

l = [I1, I2, I3, I4, I5, S1, S2, S3, S4, S5, O1, O2, O3, O4, O5, B1, B2, B3, B4, B5, W11, W22, W33, W44, W55, W21, W31, W32, W41, W42, W43, W51, W52, W53, W54, WB21, WB31, WB32, WB41, WB42, WB43, WB51, WB52, WB53, WB54]
xi = numpy.linspace(0, 0, dataNum)

def initial():

    for obj in l:
        for i in range(dataNum):
            obj[i] = 0

    S1[0] = c
    S2[0] = c
    S3[0] = c
    S4[0] = c
    S5[0] = c
    for i in range(0, dataNum):
        xi[i] = i

def reaction1(A, B, C, D, E, k, i):
    # A + B -> C + D + E
    if i + 1 < dataNum:
        det = k * A[i] * B[i]
        A[i + 1] = (A[i] - det) if A[i + 1] == 0 else (A[i + 1] - det)
        B[i + 1] = (B[i] - det) if B[i + 1] == 0 else (B[i + 1] - det)
        C[i + 1] = (C[i] + det) if C[i + 1] == 0 else (C[i + 1] + det)
        D[i + 1] = (D[i] + det) if D[i + 1] == 0 else (D[i + 1] + det)
        E[i + 1] = (E[i] + det) if E[i + 1] == 0 else (E[i + 1] + det)

def reaction2(A, B, C, D, k, i):
    # A + B -> C + D
    if i + 1 < dataNum:
        det = k * A[i] * B[i]
        A[i + 1] = (A[i] - det) if A[i + 1] == 0 else (A[i + 1] - det)
        B[i + 1] = (B[i] - det) if B[i + 1] == 0 else (B[i + 1] - det)
        C[i + 1] = (C[i] + det) if C[i + 1] == 0 else (C[i + 1] + det)
        D[i + 1] = (D[i] + det) if D[i + 1] == 0 else (D[i + 1] + det)

def reaction_parallel(Input, start, end):
    if start != 0:
        start -= 1
    Input[start] = n * c
    for i in range(start, end):
        # Release Reaction
        reaction1(I1, S1, O1, B1, W11, k1, i)
        reaction1(I2, S2, O2, B2, W22, k2, i)
        reaction1(I3, S3, O3, B3, W33, k3, i)
        reaction1(I4, S4, O4, B4, W44, k4, i)
        reaction1(I5, S5, O5, B5, W55, k5, i)

        # Inhibite Reaction
```

```
        reaction2(I2, S1, B1, W21, k1, i)
        reaction2(I3, S1, B1, W31, k1, i)
        reaction2(I3, S2, B2, W32, k2, i)
        reaction2(I4, S1, B1, W41, k1, i)
        reaction2(I4, S2, B2, W42, k2, i)
        reaction2(I4, S3, B3, W43, k3, i)
        reaction2(I5, S1, B1, W51, k1, i)
        reaction2(I5, S2, B2, W52, k2, i)
        reaction2(I5, S3, B3, W53, k3, i)
        reaction2(I5, S4, B4, W54, k4, i)

        # Kill Reaction
        reaction2(B2, S1, B1, WB21, k1, i)
        reaction2(B3, S1, B1, WB31, k1, i)
        reaction2(B3, S2, B2, WB32, k2, i)
        reaction2(B4, S1, B1, WB41, k1, i)
        reaction2(B4, S2, B2, WB42, k2, i)
        reaction2(B4, S3, B3, WB43, k3, i)
        reaction2(B5, S1, B1, WB51, k1, i)
        reaction2(B5, S2, B2, WB52, k2, i)
        reaction2(B5, S3, B3, WB53, k3, i)
        reaction2(B5, S4, B4, WB54, k4, i)

def reaction_orders(Input1, Input2, Input3, Input4, Input5, time, isOpen = False):
    initial()
    start = 0
    end = time
    reaction_parallel(Input1, start, end)

    start = time
    end = 2 * time
    reaction_parallel(Input2, start, end)

    start = 2 * time
    end = 3 * time
    reaction_parallel(Input3, start, end)

    start = 3 * time
    end = 4 * time
    reaction_parallel(Input4, start, end)

    start = 4 * time
    end = dataNum
    reaction_parallel(Input5, start, end)

    if isOpen:
        openall(xi)

    # showALlData(dataNum)
    show()

def show():
    ax=plt.gca()
    ax.spines['top'].set_linewidth(2)
```

```
    ax.spines['bottom'].set_linewidth(2)
    ax.spines['left'].set_linewidth(2)
    ax.spines['right'].set_linewidth(2)

    plt.xlabel('Time(S)', fontsize=20)
    plt.ylabel('Concentration(NM)', fontsize=20)

    plt.plot(xi, O1, color="green", label="O1", linewidth=6)
    plt.plot(xi, O2, color="orange", label="O2", linewidth=6)
    plt.plot(xi, O3, color="blue", label="O3", linewidth=6)
    plt.plot(xi, O4, color="red", label="O4", linewidth=6)
    plt.plot(xi, O5, color="black", label="O5", linewidth=6)

    plt.tick_params(labelsize=20)
    plt.legend(fontsize=20)
    plt.show()

flag = False
# reaction_orders(I2, I3, I1, I5, I4, 40, flag)
# reaction_orders(I3, I2, I5, I4, I1, 40, flag)
# reaction_orders(I1, I3, I5, I4, I2, 40, flag)
reaction_orders(I2, I4, I3, I5, I1, 40, flag)
```
